# Immunogenicity and safety of polio vaccines in infants: a systematic review of randomized clinical trials

**DOI:** 10.1186/s12985-025-02977-3

**Published:** 2025-10-29

**Authors:** Roman P. Terekhov, Artem A. Svotin, Maria D. Korochkina, Anastasiya A. Khodyachikh, Mikhail A. Varnavskiy, Anastasia N. Piniaeva, Yury Yu. Ivin, Dmitry D. Zhdanov, Liubov I. Kozlovskaya, Amir Taldaev

**Affiliations:** 1 Nelyubin Institute of Pharmacy, Sechenov First Moscow State Medical University, Trubetskaya Str. 8/2, Moscow, 119991 Russia; 2 Chumakov FSC R&D IBP RAS (Institute of Poliomyelitis), Village of Institute of Poliomyelitis 8, Building 1, Intra-City Area Municipal District Filimonkovskiy, Moscow, 108819 Russia; 3 Institute of Biomedical Chemistry, Pogodinskaya Str. 10/8, Moscow, 119121 Russia; 4 Department of Biochemistry, Peoples’ Friendship University of Russia named after Patrice Lumumba (RUDN University), Miklukho-Maklaya St. 6, Moscow, 117198 Russia; 5 Institute of Translational Medicine and Biotechnology, Sechenov First Moscow State Medical University, Trubetskaya Str. 8/2, Moscow, 119991 Russia; 6 Research Center for Molecular Mechanisms of Aging and Aging-Related Diseases, Moscow Center for Advanced Studies, Kulakova Str. 20, Moscow, 141700 Russia; 7 Shemyakin-Ovchinnikov Institute of Bioorganic Chemistry RAS, GSP-7, Miklukho- Maklaya Str. 16/10, Moscow, 117997 Russia

**Keywords:** Poliomyelitis, Vaccines, Immunization, Immunogenicity, Infants, Systematic review, Randomized clinical trials

## Abstract

Poliomyelitis, preventable only through vaccination, remains a global health concern, with wild poliovirus transmission and the emergence of vaccine-derived polioviruses. The risk of further deterioration of the situation jeopardizes efforts to eradicate polio, which has been a long-term goal for the whole world. In this systematic review, an analysis of randomized clinical trials was carried out to comprehensively assess the immunogenicity and safety of various polio immunization methods in infants. Geometric mean neutralizing antibody titers (GMT) data collected after 28–31 days after immunization were used to calculate the geometric mean titer ratio (GMR), the analysis of which showed that both inactivated polio vaccine (IPV) and Sabin strain-based inactivated polio vaccine (sIPV) as primary vaccination induce high antibody rates. Average GMR rates (CI = 0.05) for the 3 types of polio were 83.08, 33.60, and 166.30 for IPV and 234.35, 44.04, and 163.13 for sIPV, with fractional IPV showing similar results. One or two doses of IPV were insufficient to induce protection levels of antibodies against type 2 poliovirus. The novel oral polio vaccine type 2 (nOPV2) and trivalent oral polio vaccine (tOPV) also demonstrated immunogenicity in establishing immunity comparable to the inactivated vaccine; the latter exhibited an average GMR of 50.75 for serotype 2. High antibody levels were also induced by combined vaccine schedules, with sIPV-sIPV-bOPV (GMR of 1,172.7 for type 1 and 887.6 for type 3) and IPV combinations with diphtheria-tetanus-whole-cell pertussis, hepatitis B and *Haemophilus influenzae* type b (351.2, 258.8, and 573.6 for the 3 types) or pentavalent rotavirus vaccine (354.6, 117.7, and 540.9 for the 3 types) establishing particularly high antibody levels. Analysis of adverse events presented all vaccines to be well-tolerated and safe, with a tendency for combination vaccines to have a higher frequency of local reactions and fever. While the studies presented a diverse landscape with some existing areas of concern, this review provides structured evidence supporting the safety and immunogenicity of existing polio vaccines, as well as highlighting the interchangeability of different vaccination approaches in infants. Future research should aim to provide detailed reporting of adverse events in order to facilitate more comprehensive assessment of vaccine immunogenicity and, therefore, efficacy.

**Supplementary Information:**

The online version contains supplementary material available at 10.1186/s12985-025-02977-3.

## Introduction

Poliomyelitis is a highly contagious disease that can be contracted by an unvaccinated person of any age, but it more often occurs in children under 5 years of age. The causative agent of this disease is poliovirus, a small, non-enveloped virus containing a single-stranded positive-sense RNA genome, about 7,500 nucleotides long, enclosed in a protein capsid. The capsid is made up of 60 protomers composed of four viral proteins (VP 1–4) arranged in icosahedral symmetry, with VP 1–3 creating antigenic sites for receptors and antibodies to bind. Based on the slight differences in the capsid structure [1, 2], three types of polioviruses (types 1, 2 and 3) are distinguished [3, 4].

There is no cure for polio, and the nerve damage and paralysis that it causes can be permanent or even fatal [5]. The only way of preventing the disease is through vaccination, which has proven to be effective against all 3 types of poliovirus. The two main forms of polio vaccine are oral polio vaccine (OPV) and inactivated polio vaccine (IPV) [6, 7]. There are different variations of these vaccines: the conventional inactivated polio vaccine made with killed wild-polio types (Salk-IPV, IPV), inactivated vaccine made from Sabin live-attenuated (weakened) strains (sIPV), IPV with dose reduction through adsorption to aluminium hydroxide (aluminium hydroxide-adjuvanted) (IPV-Al), monovalent polio vaccine protective against the 2 type polio (mOPV2) and a novel monovalent OPV (nOPV2), trivalent oral poliovirus vaccine containing all 3 strains (tOPV), and bivalent (contains only type 1 and type 3 Sabin strains) oral poliovirus vaccine (bOPV).

Both OPV and IPV have been widely applied since the 1950–60s and continue to be a crucial part of the Global Polio Eradication Initiative (GPEI) – since its launch in 1988, polio incidence has globally decreased by over 99%, leaving only the wild polioviruses type 1 still endemic in Pakistan and Afghanistan [5, 8, 9].

While OPV is effective in inducing intestinal immunity and is generally safe, it is crucial to acknowledge its potential risks. The live-attenuated polioviruses within OPV, in rare cases, can cause vaccine-associated paralytic poliomyelitis (VAPP). Furthermore, these attenuated viruses can mutate and evolve into circulating vaccine-derived polioviruses (cVDPV), which are capable of causing polio cases and outbreaks [10–12]. Notably, polio vaccine type 2 is most frequently associated with VDPV2 and is the primary cause of most polio outbreaks linked to vaccination. For this reason, a global switch from trivalent OPV types 1, 2, and 3 to bivalent OPV types 1 and 3 was performed in 2016. This process included a transition to vaccination schedules that include IPV to maintain immunity to polioviruses type 2. Moreover, IPV contains an inactivated virus and carries no risk of VAPP or VDPV [13–17].

Routine childhood immunizations, including a polio vaccination, are typically administered 4 times total at 2, 4, 6 months, and between 4 and 6 years of age. These scheduled immunizations are designed to provide robust protection against a range of preventable diseases. The GPEI operates alongside these routine programs, conducting supplementary immunization activities (SIAs) to reach underserved populations and interrupt poliovirus transmission in high-risk areas. The GPEI aims to expand routine immunization in developing countries. It is important to introduce any immunization everywhere in order to establish primary immunization schedules.

Due to the increasing cost of GPEI, the question of its necessity arises, given the low number of polio cases and the nearly global routine immunization. Nevertheless, continuing the program is critical, and not only to ensure vaccination coverage, but also because it is a cost-effective decision with substantial long-term benefits that far outweigh the expenses. The cost of health care saved by preventing polio and related cases of paralysis is more than two to three times higher than the cost of polio eradication in the long term. Over the years 1970–2050, polio vaccination will prevent about 42 million polio cases, and thus prevent about 855,000 deaths and more than 4 million paralysis cases [18]. In terms of disability-adjusted life years (DALYs), vaccination and polio eradication will save about 39.5 million years by the year 2050 [18, 19].

In recent years, low immunization coverage hinders the GPEI progress. Between 2021 and 2025 there were 2,560 paralysis cases recorded in regions such as parts of Africa, Yemen, Indonesia and other countries, attributed to newly formed cVDPVs of all three types [20]. Moreover, in Afghanistan and Pakistan, endemic transmission of wild poliovirus type 1 (WPV1) continues with 24 confirmed cases in the area in 2025 [10, 20, 21].

Moreover, this decline in vaccination has been intensified by numerous cases of misinformation and anti-vaccine activism that have emerged in the last couple of years. The most common misinformation pieces claimed that vaccines were unsafe and that they could cause other diseases. Battling such accusations by providing grounded proof is necessary, and is now more urgent than ever, as the GPEI sets 2026 as the year for complete polio eradication.

The possibility of virus breakouts and further spread of misinformation on a wider scale is putting at risk the GPEI goal. Hence, it is extremely important to maintain high vaccination rates and to raise awareness of the importance of vaccination. Which is why the aim of this review is to provide a comprehensive summary of data regarding various immunization methods to demonstrate the immunogenicity and safety of existing polio vaccines.

## Materials and methods

### Study design

The design of this systematic review was conducted by the Preferred Reporting Items for Systematic Reviews and Meta-Analyses (PRISMA) guidelines [22]. A protocol for this study has been registered on PROSPERO [23] under ID CRD42024574830 in August 2024.

### Data sources

A systematic search of papers was conducted using the following databases: PubMed/MEDLINE, Google Scholar, and eLibrary.

### Search strategy

The search strategy in English combined the following terms: (poliomyelitis OR (infantile AND paralysis) OR “essential paralysis” OR “Heine-Medin disease” OR tephromyelitis OR polio) AND (immunogen* OR efficacy OR seroconversion) AND (vaccin* OR poliovaccine). In Russian the equal terms were used.

### Inclusion and exclusion criteria

The inclusion and exclusion criteria were based on PICOS principles [24]. They are summarized in Table 1.

**Table 1:** Inclusion and exclusion criteria

Criteria	Inclusion	Exclusion
Population	Infants (age from birth to 1 year) Males or females Healthy	Any illness or medical condition
Intervention	Immunization by any polio vaccine	Absence of immunization by any polio vaccine
Control	The presence of control group in analyzed publications is not necessary	
Outcome measure(s)	Geometric mean neutralizing antibody titers (GMT) before immunization and at the day 21–31 after immunization Data separated depending on the polio serotype	No GMT data before immunization No GMT data at the day 21–31 after immunization No serotype details for GMT data
Study design	Randomized clinical trials	Study of any other design

Only papers that provided the full text in English or Russian were included in the systematic review.

### Data collection

All papers obtained from the databases were recorded in a cloudified table. Duplicates were removed prior to screening. Two researchers (A.A.Kh. and M.A.V.) independently evaluated the article set according to including and excluding criteria, based on the titles and abstracts. In case of disagreement, the third researcher (Yu.Yu.I.) served as a referee. Then full texts of selected studies were assessed for eligibility.

### Data extraction

Two reviewers (A.A.Kh. and M.A.V.) independently performed the data extraction. The following data were in focus: number of immunized patients, country of study; dose, timing, formulation, administration, combination, and valency of vaccine; antibody titer; side effects (number of cases), poliomyelitis cases (number of cases); bibliographical details.

### Data analysis

Three researchers (R.P.T., A.A.S., and A.A.Kh.) performed the formal analysis of extracted data. To standardize the immunogenicity value of polio vaccines in selected studies, the GMR was calculated, using the following formula:

1 $$\:GMR=\:\frac{{GMT}_{n}}{{GMT}_{0}}$$

where GMT_0_ is the GMT value before the immunization and GMT_n_ is the GMT value on the day 21–31 after the immunization.

The obtained GMR values, number of side effect cases, and the number of participants were combined based on the types of vaccines and patients’ characteristics. To evaluate the significance of differences between the groups in the analyzed parameters, the mean values and the confidence intervals were calculated (*p* = 0.05).

### Risk of bias assessment

The included studies were independently assessed by the two researchers (M.D.K. and A.N.P.), using the version 2 of the Risk of Bias (RoB 2) tool [25]. The quality of evidence for the primary outcomes was evaluated via the following bias domains: randomization process, deviations of intended interventions, missing outcome data, measurement of the outcome, and selection of the reported results. In case of discrepancies, they were resolved by a tiebreaker reviewer (L.I.K.). To visualize the results of the RoB assessment, the ROBVIS tool was applied [26]. To evaluate the publication biases, the funnel plots were built in case of enough volume of data (*n* ≥ 10).

## Results

### General outlook on the scientific landscape

**Fig. 1:**
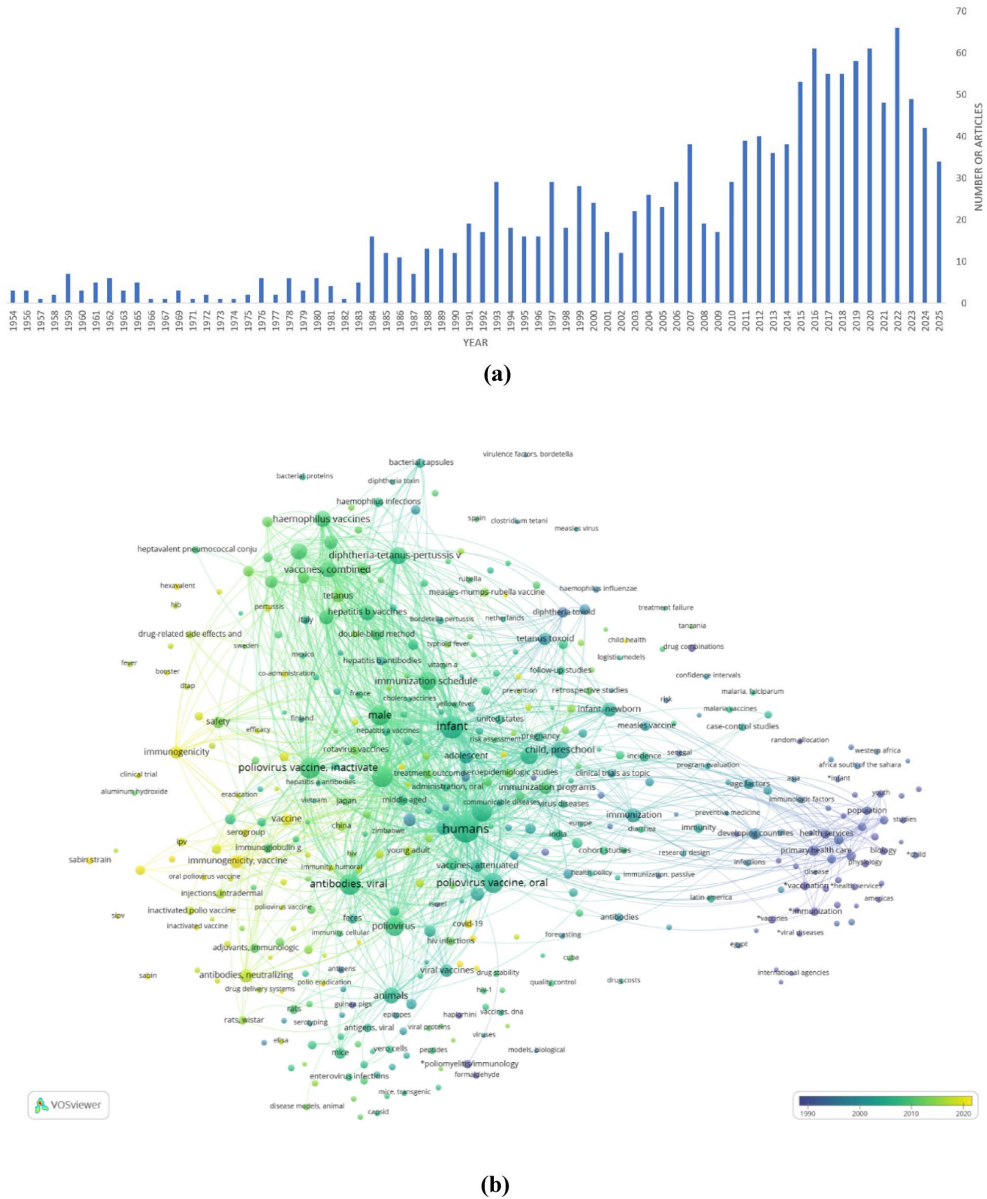

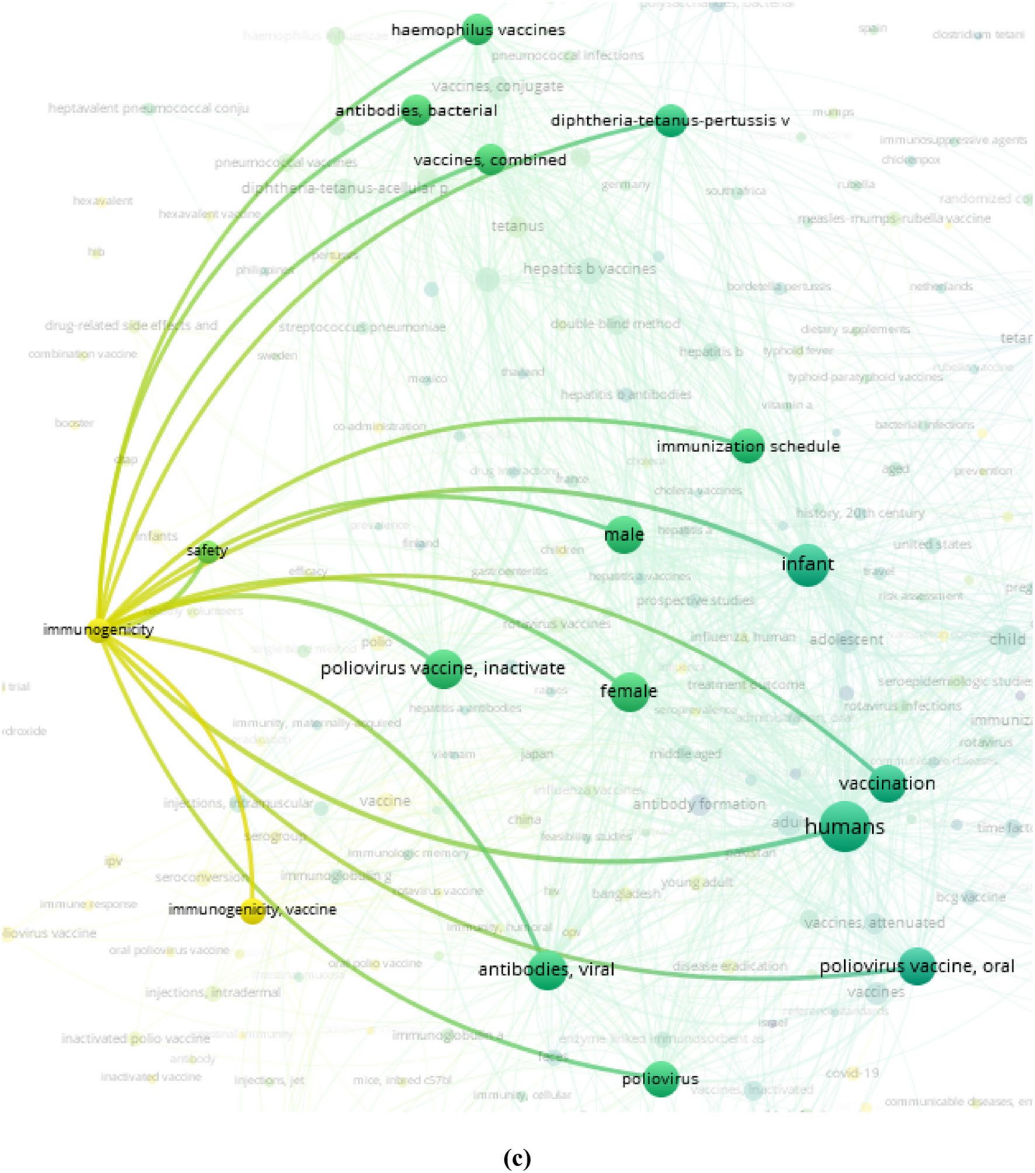
**(a)** Article count trend; **(b**,** c)** Bibliometric network of terms “poliovirus” and “vaccine”: **(b)** general view of network; **(c)** co-occurrence terms for “immunogenicity”. Created by VOSviewer [28–30]

Since 1984, the number of articles on polio vaccines has visibly increased. Over the past 10 years, the number of articles has reached about 60 on average per year (Fig. 1a). These data deposited in PubMed were used to build a bibliometric network based on keywords co-occurring with the terms “poliovirus” and “vaccine” in articles (Fig. 1b). The terms “humans”, “infant”, “male”, “female”, “poliovirus vaccines, inactivate”, “poliovirus vaccines, oral”, and “antibodies, viral” are the most frequently used, which is indicated by their bubble size.

We also looked at the general publication trends of the twentieth century, and it was the middle of the 1980 s when the number of studies on polio vaccines began to increase. The overall scientific background at the time did not experience any sudden changes while continuously growing since the late 1940 s [27]. It is also noteworthy, that there has been a shift in interest towards the immunogenicity of the polio vaccine in recent years (Fig. 1c) that confirms the significance of the systematic review.

### Process of collection and selection of the studies

**Fig. 2:**
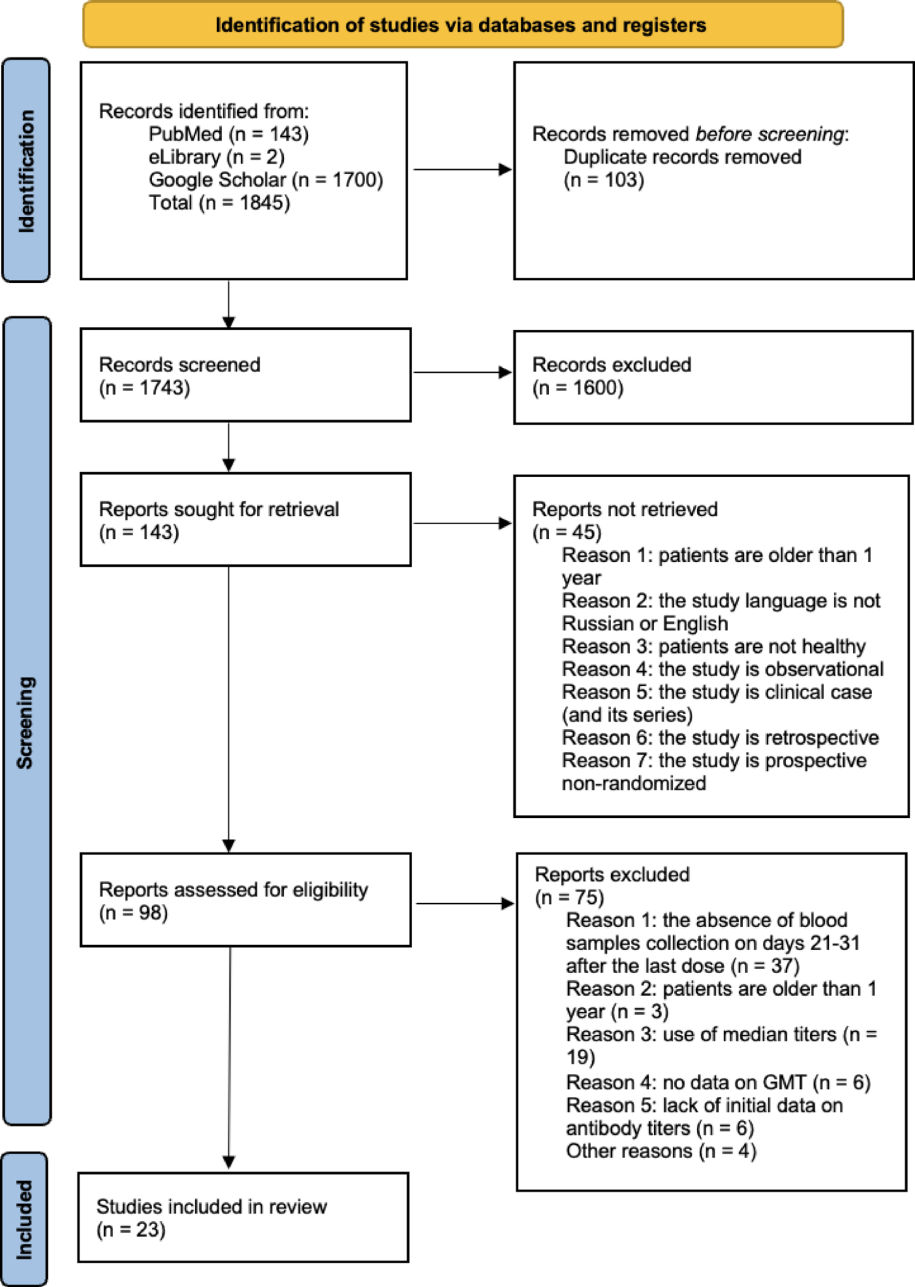
PRISMA flowchart of the search and selection process of the articles

A total of 1,845 articles were identified through the search strategies of Pubmed/MEDLINE, eLibrary and Google Scholar. Before the screening, 102 duplicates were removed. After the first screening, 1,600 articles did not meet the inclusion criteria based on their titles and abstracts, and therefore were excluded. After the second screening, an additional 48 articles were removed. During the final screening, 75 records were excluded for the following reasons: the absence of blood sample collection on days 21–31 after the last vaccination (*n* = 37), patients were older than 1 year of age (*n* = 3), use of median titers (*n* = 19), no data on GMT (*n* = 6), lack of initial data on antibody titers (*n* = 6), other reasons (*n* = 4). A total of 23 studies was included in this systematic review. The collection and selection process are illustrated in the PRISMA flow diagram (Fig. 2).

### Qualitative synthesis

A total of 15,052 participants were analyzed in the 23 included studies. The studies were conducted in China, Dominican Republic, Finland, Panama, Japan, India, Bangladesh, South Korea, Philippines, Thailand, Gambia, Taiwan, and Singapore. 1,065 infants were assigned to the groups administered only IPV (Salk vaccine) [31–35], another 969 infants to the groups which received IPV-Al (aluminium hydroxide-adjuvanted IPV) [31, 36] as part of their immunization. A number of studies included administration of sIPV (IPV from Sabin strains) to a total of 3,406 infants [32, 35, 37–39]. The efficacy of OPV (nOPV2, bOPV, tOPV) against polio strains was assessed in 3,738 participants [33, 34, 40–42]. One of the studies evaluated the effectiveness of a combined IPV and OPV (bOPV or tOPV) vaccination with 563 recipients [34], and another trial was conducted with 374 recipients and the combination of sIPV and bOPV [37]. The majority of participants, in the number of 4,345 were administered some kind of combination of IPV [36, 43–52] with DTaP/DTwP (diphtheria-tetanus-acellular/whole-cell pertussis vaccine), Hib (*Haemophilus influenzae* type b), PRP ∼ T (polyribose ribitol phosphate conjugated to tetanus protein), HBV (hepatitis B vaccine) or RV5 (pentavalent rotavirus vaccine) [53]; combined vaccines with sIPV were administered to 592 participants [39, 43]. In a single study, 219 participants were administered a combination of DTwP-HB-Hib and bOPV with IPV [45].

The intervention method was either injection (intramuscular (IM), intradermal (ID), subcutaneous (SC)) or oral administration (*per os*). In all the studies, a full dose of OPV was administered. IPV doses varied from a full to a reduced dose (1/*n*, *n* = 3, 5, and 10) with one of the trials assessing the efficacy of a low, medium, and high dose.

The findings from the 23 articles are summarized in Supplementary Table 1. For the same vaccines administered in more than 1 study, the average GMR was calculated (95% CI) and presented in Table 2. Due to the lack of clarification on the exact vaccines administered in studies with IPV or sIPV when trial subjects were allowed to be vaccinated with “other vaccines” according to the routine immunization schedule, these groups were not included in the table. Also, groups with no possibility to calculate confidential intervals were not included.

**Table 2:** Statistical processing of data from several articles

Type of polio vaccine	Sample size	Dosage form	Average GMR, IU					
			Serotype I		Serotype II		Serotype III	
			GMR	SR	GMR	SR	GMR	SR
IPV	1,065	INJ 1.0	83.08 ± 86.90	96.92 ± 3.07	33.60 ± 18.78	93.06 ± 5.90	166.30 ± 109.12	98.44 ± 1.30
sIPV	3,406	INJ 1.0	234.35 ± 175.25	98.05 ± 1.77	44.04 ± 12.58	96.43 ± 2.74	163.13 ± 76.94	98.57 ± 1.42
tOPV	374	Oral 1.0	257.55 ± 203.15	98.08 ± 3.77	50.75 ± 28.13	98.72 ± 2.51	88.05 ± 13.03	99.15 ± 1.67
DTaP-IPV/Hib	651	INJ 1.0	129.27 ± 61.72	–	83.84 ± 17.42	–	247.87 ± 49.98	–
DTPa-HBV-IPV/Hib	510	INJ 1.0	36.45 ± 39.10	–	42.50 ± 32.54	–	152.10 ± 71.73	–

*Data are presented as average GMR/Seroconversion rates (SR) ± half-width of the confidence interval (*p* < 0.05)

### Risk of bias

Possible forms of bias were assessed according to the RoB 2 tool. Overall, 2 of the 23 studies showed a high risk of bias and 8 others raised some concerns.

**Fig. 3:**
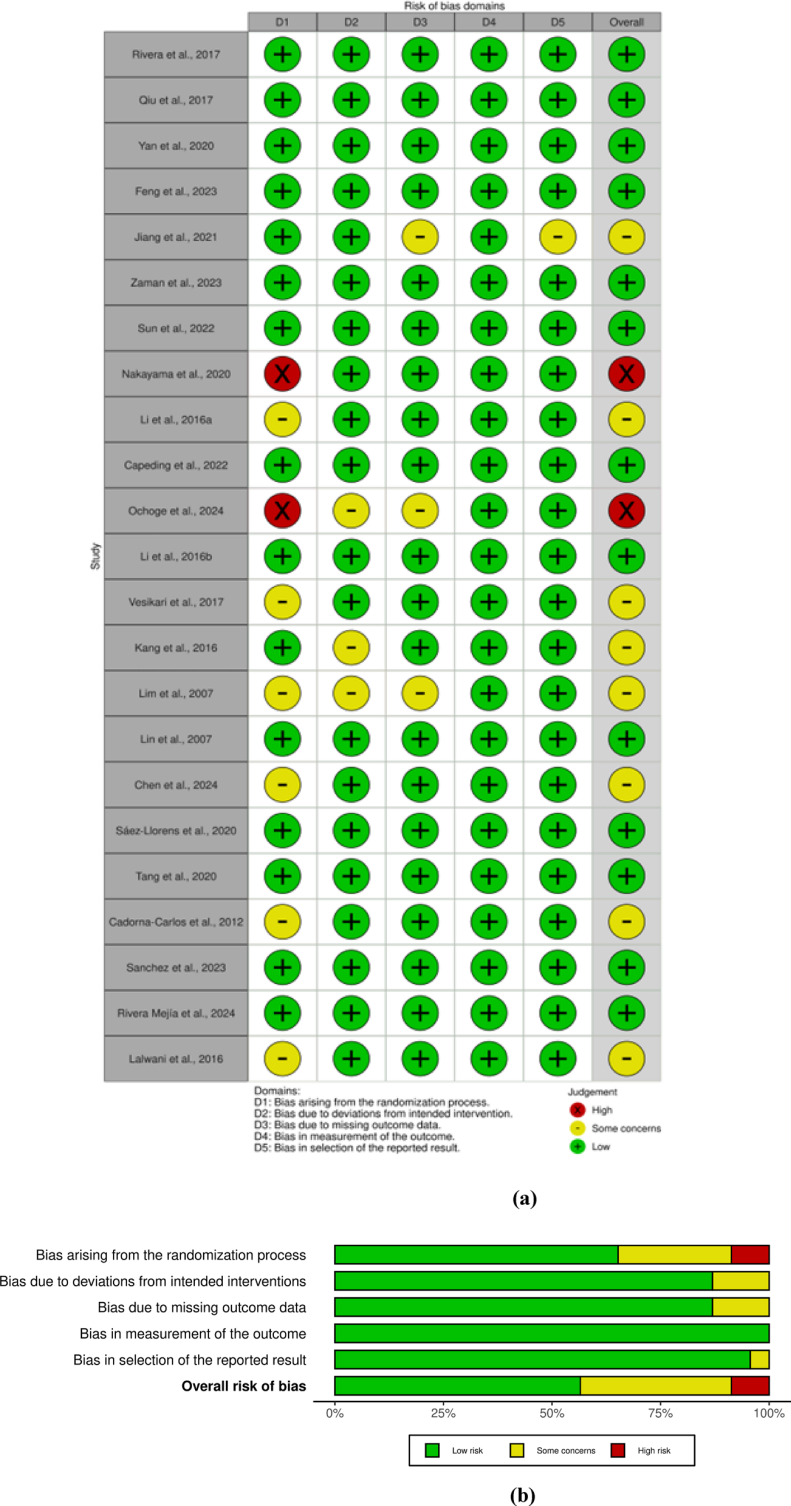
**(a)** Risk of bias summary “traffic light”; **(b)** Weighted bar plot

A “traffic light” summary of the “low”, “high”, or “some concerns” risk of bias assessment of the included studies via 5 domains is presented in Fig. 3a.

The weighted bar plot (Fig. 3b) provides a graphical representation of the distribution of risk-of-bias judgments within each bias domain. Since the judgments are weighted, the segments represent the proportional distribution of studies categorized as “low”, “some concerns”, and “high.” Consequently, the overall risk-of-bias bar indicates an increase in the percentage of studies categorized as “some concerns,” reflecting the consideration of various types of biases and the relative importance of each study.

### Immunogenicity

**Fig. 4:**
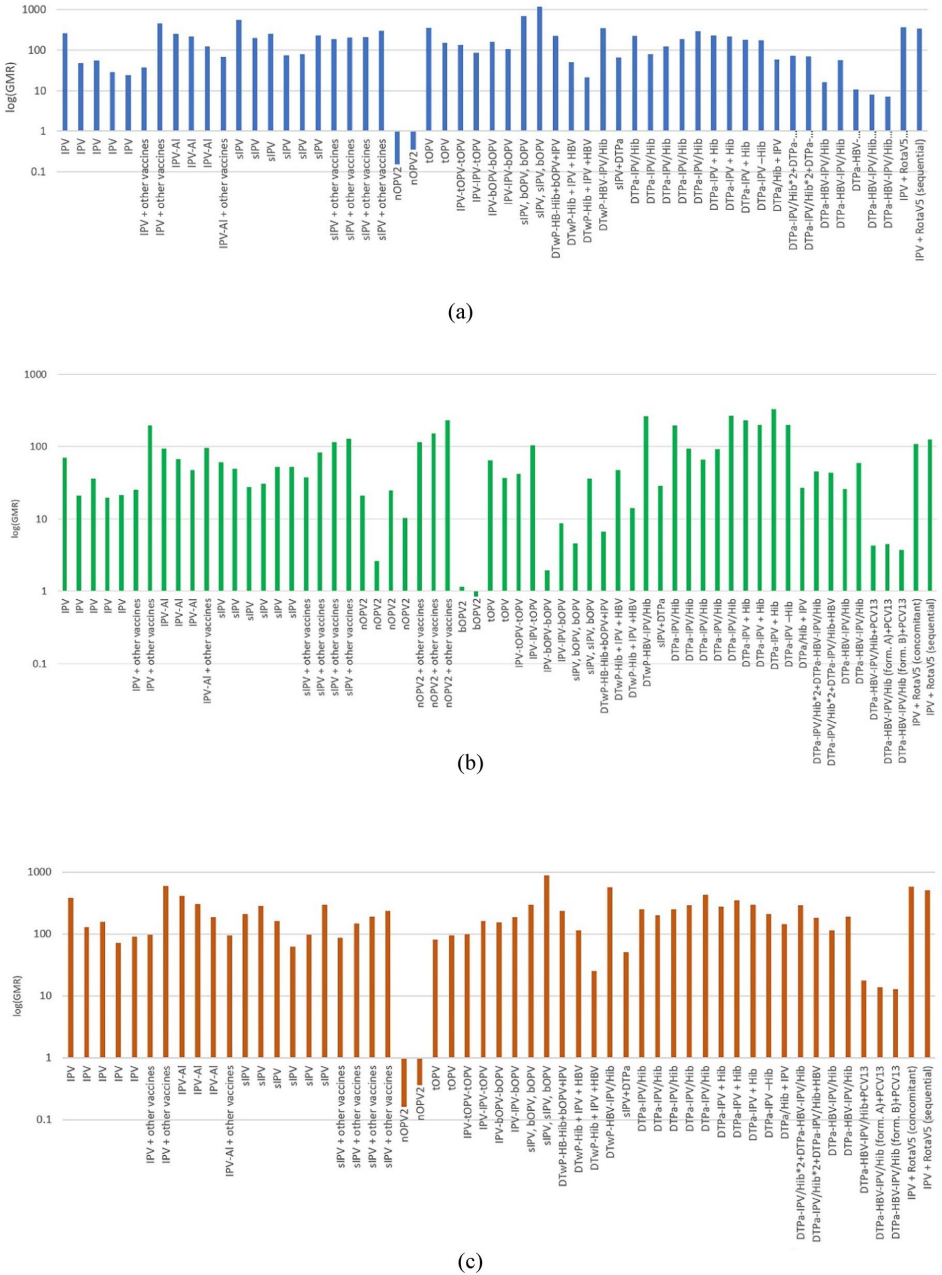
Logarithmic scales of GMR data for serotypes: **(a)** serotype 1, **(b)** serotype 2, **(c)** serotype 3

Overall, the majority of the studies reported on the immunogenicity of different types of vaccine against the three poliovirus strains (Sabin 1–3). This was confirmed by the increase of antibodies after complete immunization programs and evident from the GMR ranging from 7.1 to 1,172.7 for serotype 1, from 2.0 to 327.2 for serotype 2 and from 12.8 to 590.7 for serotype 3 (titers below 8 are considered non-protective, i.e. negative). These data are presented in Fig. 4 as logarithmic scales for each serotype

**Fig. 5:**
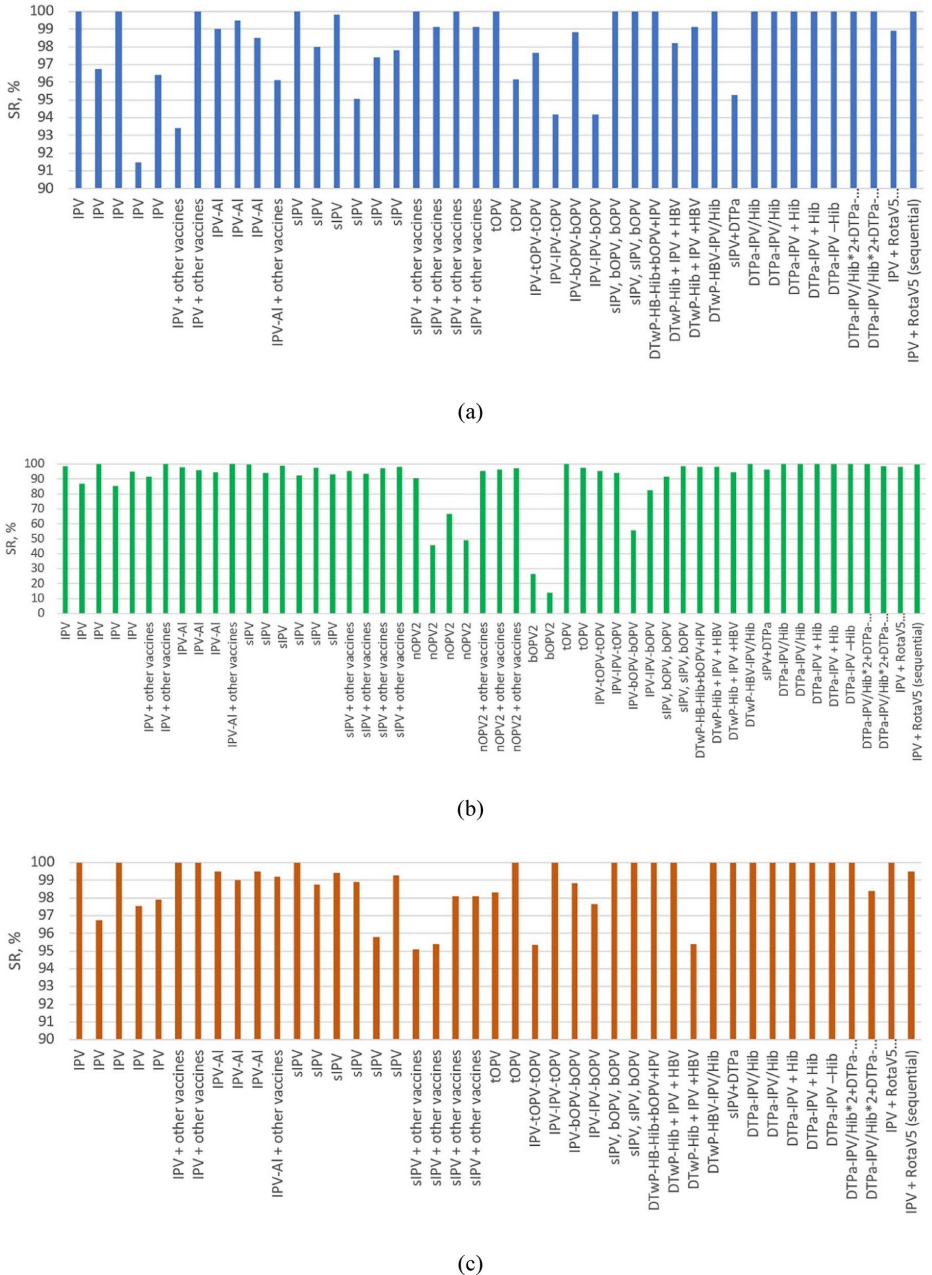
SR data for serotypes: **(a)** serotype 1, **(b)** serotype 2, **(c)** serotype 3

SR (considered positive at antibody titer ≥ 4 times higher than the estimated maternal antibody titer and a titer ≥ 8, one month after the primary vaccination series) consistently varied from 91.5 for serotype 1 and from 95.1 to 100.0 for serotype 3. For serotype 2, the lowest values were 45.7, 49.0, and 66.7. The data are summarized in Fig. 5. Full information is presented in Supplementary Table 1.

Primary immunization with 3 IPV doses showed generally high GMRs and SR rates, with sIPV groups exhibiting similar values. Likewise, fractional IPV doses (1/10) exhibited the same immunogenicity as full doses, in some IPV-Al (IM) groups demonstrating even higher values. Comparison of the GMTs in groups administered (s)IPV as opposed to OPV showed that a single dose of nOPV2 as primary or 1–2 doses as boost immunization is not enough to build immunity against PV2 (SR = 45.7, 49.0 for primary and 66.7 for boost). However a full primary vaccination with tOPV or nOPV2 exhibits GMRs and SR against PV2 similar to (s)IPV. This indicates that all the above-mentioned vaccination schedules are interchangeable and effective.

The peak results were recorded among schedules of sIPV with bOPV and combinations of IPV with vaccines against other diseases (DTaP, PRP etc.); sIPV-sIPV-bOPV demonstrated the highest antibody levels and 100% seroconversion for serotype 1 and 3, with the GMRs being 1,172.7 and 887.6, respectively. The highest GMR value for serotype 2 was shown by the DTPa-IPV + Hib and is equal to 327.2 (SR also 100%). IPV + RV5 and DTwP-IPV-HB-PRP ~ T had one of the highest GMR scores for all three serotypes (354.6, 117.7, 540.9 and 351.2, 258.8, 573.6, respectively).

It is important to note that the lowest GMRs against type 2 were reported in the sIPV-bOPV-bOPV and IPV-bOPV-bOPV groups (4.6 and 2.0) and D_A_T_A_Pa-HBV-IPV/Hib and D_B_T_B_Pa-HBV-IPV/Hib groups with values ranging from 3.7 to 13.8 between the 3 serotypes. Both the D_A_T_A_Pa-HBV-IPV/Hib and D_B_T_B_Pa-HBV-IPV/Hib groups belong to the same study, as does the 238 participants DTPa-HBV-IPV/Hib group, that also had low GMR rates in comparison to groups that were administered the same vaccine but were part of other studies.

When it comes to the IPV-bOPV-bOPV group, seroconversion for type 2 was unusually low – only 55.8%. Moreover, in the IPV-IPV-bOPV group, post-vaccination values were also rather low (GMT = 8.8 and seroconversion = 82.6), hence it is clear that one or two doses of IPV in primary immunization is insufficient for inducing immunity to PV 2.

### Safety assessment

An important part of this systematic review was the assessment of the safety of vaccines. The main findings are as follows: Overall, the vaccines were considered well-tolerated and safe. However, some articles highlighted the need for further research. The most common adverse events (AEs) included fever (pyrexia), abnormal crying, diarrhea, vomiting, drowsiness, irritability, and injection site reactions (sensitivity (tenderness), pain or erythema, swelling). Less common AEs included pneumonia, upper/lower respiratory tract infections, and bronchiolitis. The most frequently reported AEs were generally consistent with the typical side effects of vaccination [54].

Overall, all individual vaccines demonstrated a satisfactory level of safety in the conducted studies. When different individual vaccine types (IPV, sIVP, or tOPV) were compared in a single study, no significant differences were found between the study groups, indicating a similar safety profile.

For different doses of IPV-Al there was either an extensive list of AEs, or a complete lack of data. Hence, more research is needed to gather data on the safety of aluminum-adjuvanted polio vaccines, given that they provide a strong immune response, are widely used, and are considered to be safe [55].

AE data for IPV-bOPV-bOPV did not contain any differences in comparison to similar vaccination groups; the same applies to D_B_T_B_Pa-HBV-IPV/Hib. The general trend for AEs of combinations was similar to single-component vaccines: Injection site reactions were most frequent, systemic reactions (like fever, irritability, drowsiness, crying, loss of appetite, vomiting, and diarrhea) were all common. It is important to note, that one of the fatal AE occurred in the D_A_T_A_Pa -HBV-IPV/Hib (asphyxia and interstitial lung disease) study and the other one in the DTaP-IPV/Hib (infectious shock due to acute bronchopneumonia and congestive heart failure) group. These deaths were not considered as related to the vaccine.

Among the studies, three fatal cases were recorded. The causes of death were asphyxia and interstitial lung disease for one case, septic shock for another, and infectious shock due to acute bronchopneumonia and congestive heart failure for the third. The first case occurred 17 days after the first vaccine dose, the second – three months post-dose two and the third case occurred one day after the third dose. All three fatalities were considered unrelated to vaccination.

### Baseline GMTs

Considering the geographical differences, the baseline (preprimary) GMTs did vary for each poliovirus type across the studies, with the pre-vaccination type 3 titers being considerably lower than type 1 and 2 titers. Highest baseline GMTs: Bangladesh (29.3 for ST1, 56.5 for ST2, 15.1 for ST3), India (31.9 and 53.5 for ST1, 36.2 and 32.5 for ST2, 11.9 for ST 3), Dominican Republic (53.7/47.4/46.4/51.6 for ST2, 13.0 and 12.5 for ST3). Lowest baseline GMTs: Japan (3.58 and 3.35 for ST1, 5.06 and 5.55 for ST2, 2.95/2.86/3.61 for ST3), South Korea (4.80 and 4.26 for ST1, 4.34 and 4.48 for ST3), Thailand (6.70/6.07/6.66/6.47 for ST 1). Out of the 23 studies under discussion in the present work, 9 were conducted in China. In these articles, the baseline GMTs varied from 5.55 to 21.10 for ST 1; from 5.00 to 9.76 for ST2, from 4.34 to 8.08 for ST3.

## Discussion

The increase of the number of articles on polio vaccines in the middle of the 1980s most likely correlates with the global effort to eradicate polio that had begun in 1985 with the start of the PolioPlus initiative and was followed by the launch of the GPEI in 1988. All national immunizations and polio monitoring campaigns were inevitably accompanied by a large volume of publications on these programs and their results. Clearly, the 1980s have become a turning point in the fight against polio, raising global interest in collaborative polio eradication efforts that continue to be ongoing today [56].

In order to fully estimate the relevance of the review, a primary evaluation of the PROSPERO search results on the immunogenicity of polio vaccines was performed and showed that our analysis is not only relevant due to the lack of similar articles on polio vaccine, but also unique in the variety of different vaccines that were compared and assessed.

Eight of the twenty-three included articles (35%) exhibited bias related to the randomization process. This finding is problematic, as randomization is essential for minimizing bias and ensuring comparability between treatment groups. The fact that in this domain 6 articles were categorized as having “some concerns” and 2 with “high risk of bias” suggests that this issue is rather significant.

Some of the articles raised concerns due to deviations from intended intervention and missing outcome data. Such deviations can significantly impact the validity of the results, as they may lead to inconsistencies in treatment application and hinder the reproducibility of findings. Missing data further complicates interpretations and could skew results, making it difficult to assess the true effects of the interventions evaluated.

All articles except one showed a low risk of bias in selection of the reported result. With a single study displaying a risk of bias in this area, it seems to be a relative strength of the included studies. This suggests that, in most cases, researchers appear to have reported results in a transparent and unbiased manner, mitigating potential concerns of selective outcome reporting.

None of the studies were associated with bias in measurement of the outcome, indicating that once an outcome was defined, the measurements were consistently applied across studies. This stability in measurement suggests that the outcomes reported can be trusted to reflect the intended constructs reliably, although the initial deviations in intervention could still detract from overall confidence in the findings.

The risk of bias assessment presents a mixed landscape among the included studies. While certain aspects demonstrate robustness, significant concerns require careful consideration. The identification of two studies with a “high risk of bias” alongside the eight additional studies raising “some concerns” serves as a call to action for researchers in this field. It underscores the necessity for attentive methodology and transparent reporting in order to enhance the reliability of findings. This assessment is particularly timely as it paves the way for future research endeavors to focus on reducing these biases, ensuring that the resultant evidence is robust. Clearly, sIPV and IPV vaccines are the reason for the peak results in schedules of sIPV with bOPV and combinations of IPV, but the fact that they were a part of a combination schedule cannot be overlooked: Whether DTaP or PRP ∼ T had affected IPV can only be stated after additional research on the matter. In the sIPV-sIPV-bOPV study, it was probably the presence of an extra sIPV dose that led to the highest GMRs for ST 1 and 3, since sIPV-bOPV-bOPV had lower antibody rates for these serotypes.

Taking into consideration the number of participants in the groups with the lowest GMRs, it could be possible that these particular groups are not sufficient for making any conclusions on the immunogenicity of the studied vaccines. Although the wild polio type 2 is eradicated, such low antibody levels as in the IPV-bOPV-bOPV group might still pose a threat due to cVDPVs. Thus, as nOPV2 and tOPV showed very similar average GMR rates in comparison to full-dose IPV or sIPV, it is important to keep oral polio vaccine a part of the immunization [57].

Unfortunately, a comprehensive statistical comparison of AEs was not feasible due to the varying presentation of results across the publications. Some studies provided only general data on the frequency of AEs within specific groups, expressed as percentages. In contrast, other studies offered detailed summaries of individual cases of AEs occurring at different vaccination stages, categorized by specific AE types. Additionally, some articles reported data as the number of patients who developed particular AEs. This inconsistency rendered mathematical processing of the data impossible. Nevertheless, the primary trends identified through the analysis were presented above.

The analysis of AEs related to combined vaccines suggests that they tend to have higher rates of local reactions and fever compared to single-component vaccines. This outcome aligns with the main technological challenge of combined vaccines – the administration of multiple antigens simultaneously. While this increases the possibility of local adverse events, multicomponent vaccines are essential to address the growing number of vaccines required for infants and children. These vaccines have the potential to become the key part of any type of immunization against a wide range of diseases [58].

Baseline GMTs for Japan, Bangladesh, and India are very abnormal when compared to other values. Although these numbers are clearly noteworthy, it is also important to consider that the analysis included only one study from each of the countries, and more data is needed to establish a correlation between baseline GMT rates and the country of the article’s origin. The samples are too insufficient, especially in comparison to nine different articles conducted in different regions of China, all of which showed very similar GMT rates for ST2 and 3.

It is, however, important to note, that India and Bangladesh have been polio-free since 2014 – later, when compared to other countries – and are in direct proximity to Pakistan and Afghanistan, the only endemic regions. A high poverty rate in India is also consistent with pre-disposal of vaccine-preventable diseases. All in all, it is possible that these factors could have led to higher baseline GMT rates in these countries [59].

In contrast, Japan has been polio-free since 1980, largely due to its strong healthcare system, comprehensive vaccination programs, and low exposure to the virus. Low GMTs could indicate that the immunity levels in the general population are relatively high and isolation from endemic regions has reduced the risk of polio cases [60].

This analysis has some limitations that must be considered in interpreting the results. Firstly, the sample sizes in Table 1 only included participants who fully completed the vaccination, while the AE data came from all participants who received at least one vaccine dose. There was no follow-up AE information for those who finished the study, leading to inconsistencies between the sample sizes and AE numbers (see in Supplementary Table 1).

Secondly, the sample sizes among the 60 immunization groups varied greatly from 10 to 810, with an average number of participants of about 224. As a result, such a range of numbers complicated the comparison process of some vaccination groups, especially their AEs. In some cases, because the samples are so different, it is impossible to make a clear connection between the amount and diversity of side effects and the administered vaccine. Moreover, the articles used different methods of recording and presenting AE data, and some studies did not present that data in a way that was usable. Because of this, a proper comparison and evaluation of the AEs was not possible, which limited the quality of the analysis. Using standardized approaches among studies would help to generate higher-quality analyses.

Thirdly, only studies that contained GMT data were included in the review. This resulted in a nearly 5-fold decrease in the number of eligible articles, as there are several other ways to assess the immunogenicity (e.g. geometric mean concentration, median/mean titers, and vaccine immunogenicity). When measuring the immunity response induced by a vaccine, GMT is most commonly used, as it allows analyzing antibody levels in large groups by giving a good representation of skewed data [61]. That is why in this systematic review, the geometric mean titers ratio was calculated from the extracted GMT data in order to analyze the immunogenicity value of various polio vaccines and find the connections or the absence of such between different studies and their findings.

It was not in our intention to examine the longevity of immunological responses elicited by various polio vaccines, although this might have provided some valuable information concerning both OPV and IPV.

There is a possibility of inter-study differences in the measurements of antibody titers due to variations in the methods or commercial kits employed in different trials. A key reason for this is the absence of universal standards for GMT data collection. However, there are widely recognized guidelines, recommendations, and best practices from WHO, national regulatory authorities, pharmacopoeias, and other organizations, that are adhered by the researches. Based on the harmonization processes between different regulators, in this review we suggested that the results of kits’ analysis are relevant and characterized by insignificant differences in correctness.

It is also important to note that children under six months may have circulating antibodies to polio transmitted from their mothers. Therefore, the higher the polio vaccination coverage in the study region, the more antibodies a child may receive at birth. This could potentially complicate the calculation of SR.

## Conclusion

The systematic review contains an analysis and comparison of the polio vaccines and provides evidence of their immunogenicity and safety. The data is structured and presented in such a way as to provide a clear overview of all the different immunization approaches and their performances.

The study demonstrated that both IPV and sIPV are well-suited for the primary vaccination series, which includes three doses of the vaccine against poliomyelitis. However, a single or double administration is insufficient to establish stable immunity, particularly against type 2 poliovirus. It is also possible to reduce the dose of the administered antigen when using IPV-Al without compromising the levels of SR and GMR. This approach could potentially decrease the incidence of AEs and enhance compliance among patients in the context of population immunization. Additionally, despite the fact that tOPV is well-suited for establishing primary immunity, showing SR and GMR values comparable to those of IPV and sIPV, the global trend is shifting away from the utilization of live-attenuated vaccines.

In future studies on immunogenicity against poliomyelitis and, therefore, vaccine efficacy, it is essential to provide a more detailed account of the incidence of AEs, which will facilitate a more comprehensive comparison of different types of vaccines.

## Supplementary Information

Supplementary Material 1

## Data Availability

All data generated or analyzed during this study are included in this published article and its supplementary information files.
