# Supplementary material for: Immunogenicity and safety of polio vaccines in infants: a systematic review of randomized clinical trials
Source: Virol J. 2025 Oct 29;22:348. doi: 10.1186/s12985-025-02977-3 (PMC12573955; doi:10.1186/s12985-025-02977-3)
Supplement: Supplementary file 1 — Supplementary Material 1 [file 12985_2025_2977_MOESM1_ESM.pdf]

Supplementary Table S1

Immunogenicity and Safety of Polio Vaccines in Infants: A Systematic Review of Randomized Clinical Trials

Roman P. Terekhov [1,*], Artem A. Svotin [1], Maria D. Korochkina [1], Anastasiya A. Khodyachikh [1], Mikhail A. Varnavskiy [1], Anastasia N. Piniaeva [2], Yuri Yu. Ivin [2], Dmitry D. Zhdanov [3,4], Liubov I. Kozlovskaya [2,5] and Amir Taldaev [3,6,7]

[1] Nelyubin Institute of Pharmacy, Sechenov First Moscow State Medical University, Trubetskaya Str. 8/2, 119991 Moscow, Russia
[2] Chumakov FSC R&D IBP RAS (Institute of Poliomyelitis), Building 1, Village of Institute of Poliomyelitis 8, Intra-City Area Municipal District Filimonkovskiy, 108819 Moscow, Russia
[3] Institute of Biomedical Chemistry, Pogodinskaya Str. 10/8, 119121 Moscow, Russia
[4] Department of Biochemistry, Peoples' Friendship University of Russia named after Patrice Lumumba (RUDN University), Miklukho-Maklaya St. 6, 117198 Moscow, Russia
[5] Institute of Translational Medicine and Biotechnology, Sechenov First Moscow State Medical University, Trubetskaya Str. 8/2, 119991 Moscow, Russia
[6] Research Center for Molecular Mechanisms of Aging and Aging-Related Diseases, Moscow Institute of Physics and Technology, Institutsky lane 9, 141700 Dolgoprudny, Russia
[7] Shemyakin-Ovchinnikov Institute of Bioorganic Chemistry RAS, GSP-7, Miklukho-Maklaya Str. 16/10, Moscow 117997, Russia
[*] Corresponding Author: Dr. Roman P. Terekhov (email: terekhov_r_p@staff.sechenov.ru)

Vaccine	Schedule	Number of doses (M)	Form	Dose	Sample size	Immunogenicity												Rate of complications	Reference
						Serotype I				Serotype II				Serotype III					
						Before	After	GMR	SR	Before	After	GMR	SR	Before	After	GMR	SR		
IPV	Primary	3 (6-10-14W)	IM	1	206	14,4	3727,7	258,9	100	53,7	3759,2	70	98,5	11,9	4531,1	380,8	100	N/D	10.1016/S1473-3099(17)30177-9
IPV	Primary	3 (2-3-4)	IM	1	277	11,81	561,04	47,5	96,75	9,38	196,96	20,99	86,64	8,08	1044,13	129,2	96,75	Pneumonia - 15, Bronchitis - 1, Infectious diarrhoea - 1	10.1016/j.ijid.2023.01.020
IPV	Primary	3 (2-3-4)	IM	1	306	8,7	485,1	55,8	100	6,5	234,3	36,1	100	5,2	824,3	158,5	100	Diarrhoea - 1, Herpes zoster -1, Hydrocephalus -1	10.1016/j.vaccine.2016.02.010
IPV	Primary	3 (2-3-4/3-4-5)	IM	1	82	10,43	301,42	28,9	91,46	6,95	136,94	19,7	85,37	5,12	370,52	72,4	97,56	Fever - 188, Irritability - 16, Somnolence3, Vomit - 7, Diarrhea - 42, Redness on injection site - 3	10.1080/21645515.2017.1288769
IPV	Primary	3 (6-10-14W)	N/D	1	194	12,67	307,74	24,3	96,4	11,64	246,49	21,2	94,8	8,48	768,62	90,6	97,9	Pain/tenderness - 54, Erythema/redness - 13, Indurtion/swelling - 7, Fever - 10, Irritability/restlessness - 81, Drowsiness/sleepiness - 51, Loss of appetite - 29, Diarrhea - 29, Vomiting - 27, Rash - 18	10.1093/infdis/jiaa770
IPV + other vaccines	Primary	3 (2-3-4/3-4-5)	INJ	1	106	18,6	693,6	37,3	93,4	6,9	175,5	25,4	91,5	5,7	559,2	98,1	100	Redness – 15, Swelling – 2, Pain – 1, Induration – 3, Rash – 1, Pruritus – 1, Fever – 46, More crying - 28 Diarrhea – 15, Cough – 11, Vomiting – 9, Activity decline – 11, Inappetence – 5	10.1080/21645515.2020.1745593
IPV + other vaccines	Primary	3 (2-4-6)	IM	1	341	10,4	4775	459,1	100	35,7	6934	194,2	100	8,1	4785	590,7	100	Erythema - 44, Swelling – 39, Crying – 307, Pyrexia - 282, Nasopharyngitis - 248, Tonsilitis - 190, Irritability - 206, Somnolence - 129, Decreased appetite - 110, Vomiting - 83, Fever - 45, Dermatitis diaper - 43, Rhinitis - 45, Microcytic anaemia - 37, Acarodermatitis - 30, Bronchiolitis - 33, Diarrhoea - 31, Conjunctivitis - 25, Gastroenteritis - 31	10.1016/j.vaccine.2020.02.066
IPV-Al	Primary	3 (6-10-14W)	IM	/3	205	13	3310,2	254,6	99	47,4	4495,1	94,8	97,6	10,3	4229,7	410,7	99,5	N/D	10.1016/S1473-3099(17)30177-9
IPV-Al	Primary	3 (6-10-14W)	IM	/5	205	10,4	2221,1	213,6	99,5	46,4	3151,8	67,9	96,1	10,3	3120	302,9	99	N/D	10.1016/S1473-3099(17)30177-9
IPV-Al	Primary	3 (6-10-14W)	IM	/10	204	12,7	1584,6	124,8	98,5	51,6	2410,8	46,7	94,6	11,2	2069	184,7	99,5	N/D	10.1016/S1473-3099(17)30177-9
IPV-Al + other vaccines	Primary	3 (2-4-6)	IM	/10	355	11,9	809,3	68	96,1	34	3256	95,8	100	8,2	786,9	95,96	99,2	Erythema - 55, Swelling - 41, Crying - 295, Pyrexia - 259, Nasopharyngitis - 254, Tonsilitis - 206, Irritability - 192, Somnolence - 148, Decreased appetite - 113, Vomiting - 77, Temperature - 41, Dermatitis diaper - 38, Rhinitis - 33, Microcytic anaemia - 29, Acarodermatitis - Bronchiolitis - 27, Diarrhoea - 20, Conjunctivitis - 25, Gastroenteritis – 2025,	10.1016/j.vaccine.2020.02.066
sIPV	Primary	3 (2-3-4)	IM	1	195	7,93	4476,66	564,5	100	8,47	510,18	60,23	99,49	5,17	1091,66	211,2	100	Fever - 49, Rash - 3, Cough - 11, Diarrhea - 2, Running nose - 6, Redness - 2, Induration - 1, Swelling - 1	10.1016/j.vaccine.2020.07.042
sIPV	Primary	3 (2-3-4)	IM	1	810	13,61	2716,97	199,6	98	9,36	459,71	49,1	94,07	7,09	1998,23	281,8	98,77	Pneumonia - 28, Bronchitis - 11, Upper respiratory tract infection - 5, Laryngitis - 1, Conjunctivitis -1, Intracranial infection - 1, Chest wall abscess - 1, Mycoplasma infection - 1, Orchitis - 1, Herpangina - 1, Acute pulmonary edema - 1, Dyspepsia - 1	10.1016/j.ijid.2023.01.020
sIPV	Primary	3 (2-3-4 или 4-5-6)	IM	1	1096	13,02	3287,32	253,1	99,8	8,45	231,61	27,39	98,9	5,76	934,816	162,36	99,4	Redness - 193, Pain - 232, Induration - 100, Swelling - 67, Fever - 2107, Diarrhea - 445, Cough - 511, Vomiting - 427, Loss of appetite - 722, Drowsiness - 906, Irritability - 736, Abnormal crying - 1161	10.1016/j.vaccine.2021.01.027<br>10.1016/j.vaccine.2021.01.027
sIPV	Primary	3 (2-3-4)	IM	1	182	5,68	426,47	75,08	95,05	5,56	170,31	30,6	92,31	4,34	269,09	62	98,9	Fever - 24, Irritability - 10, Vomit - 9, Diarrhea - 10, Drowsiness - 6, Allergic - 2, Pain - 5, Induration - 3, Redness - 27, Swelling - 1	10.3389/fimmu.2022.905634
sIPV	Primary	3 (6-10-14W)	N/D	1	852	12,79	1016,89	79,5	97,4	13,84	732,48	52,9	97,4	10,13	995,8	98,3	95,8	Pain/tenderness - 332, Erythema/redness - 48, Induration/swelling - 38, Fever - 51, Irritability/restlessness - 403, Drowsiness/sleepiness - 258, Loss of appetite - 123, Diarrhea - 126, Vomiting - 135, Rash - 61	10.1093/infdis/jiaa770
sIPV	One dose	1 (2)	IM	1	271	12,99	3027,5	233,1	97,79	9,6	505,14	52,6	92,99	7,39	2215,44	299,8	99,26	Pneumonia - 9, Bronchitis - 1, Upper respiratory tract infection - 1, Acute pulmonary edema - 1, Intussusception - 1, Diarrhea - 1, Gastritis - 1, Capillaritis - 1	10.1016/j.ijid.2023.01.020
sIPV + other vaccines	Primary	3 (2-3-4/3-4-5)	IM	1	103	21,1	3894	184,5	100	6,9	259,4	37,6	95,1	6,5	556,9	85,7	95,1	Redness – 19, Induration – 1, Fever – 57, More crying – 25, Diarrhea - 14, Cough – 19, Vomiting – 11, Activity decline – 8, Inappetence – 5	10.1080/21645515.2020.1745593
sIPV + other vaccines	Primary	3 (2-3-4/3-4-5)	IM	LD	108	17,6	3609,9	205,1	99,1	8,4	691,2	82,3	93,5	6,2	908,2	146,5	95,4	Redness -26, Swelling – 8, Pain - 6, Induration - 9, Rash - 3, Pruritus - 1, Fever - 60, More crying - 27, Diarrhea -16, Cough - 16, Vomiting – 12, Activity decline - 4, Inappetence - 7	10.1080/21645515.2020.1745593
sIPV + other vaccines	Primary	3 (2-3-4/3-4-5)	IM	MD	105	17,5	3694,9	211,1	100	6,9	801,7	116,2	97,1	5,4	1030,1	190,8	98,1	Redness – 28, Swelling – 8, Pain – 8, Induration – 4, Rash – 2, Fever – 60, More crying – 35, Diarrhea – 10, Cough – 18, Vomiting – 9, Activity decline – 5, Inappetence – 5	10.1080/21645515.2020.1745593
sIPV + other vaccines	Primary	3 (2-3-4/3-4-5)	IM	HD	106	17,4	5252,9	301,9	99,1	7	890,2	127,2	98,1	5,7	1347,7	236,4	98,1	Redness – 35, Swelling – 6, Pain – 9, Induration - 6, Rash – 1, Pruritus – 1, Fever – 56, More crying - 34 Diarrhea – 19, Cough – 18, Vomiting – 12, Activity decline – 11, Inappetence – 10	10.1080/21645515.2020.1745593
	Primary	2 (0-1)	PerOs	1	219	29,3	4,4	0,15		56,5	1180	20,9	90,3	15,1	2,5	0,16	N/D		
nOPV2	Primary	1	PerOs	1	220	29,3	10,3	0,35		56,5	146,8	2,59	45,7	15,1	5,7	0,37	N/D	Nasopharyngitis - 27, Upper respiratory tract infection - 24, Respiratory tract infection - 23, Pneumonia - 9, Omphalitis - 7, Ophthalmia neonatorum - 6, Tinea capitis - 4, Diarrhoea -2, Abdominal distension - 2, Pyrexia - 6, Dermatitis contact - 2	10.1016/S0140-6736(22)02397-2
	Boost	2	PerOs	1	650	N/D	N/D	N/D	N/D	26,7	654,9	24,6	66,7	N/D	N/D	N/D	N/D	N/D	10.1016/S0140-6736(23)02844-1
nOPV2	Boost	1	PerOs	1	1946	N/D	N/D	N/D	N/D	26,7	277,9	10,4	49	N/D	N/D	N/D	N/D	N/D	10.1016/S0140-6736(23)02844-1
nOPV2 + other vaccines	Primary	2 (1 week)	PerOs	1	289	N/D	N/D	N/D	N/D	10,7	1232	115,1	95,2	N/D	N/D	N/D	N/D	Abnormal crying – 88, Diarrhoea – 45, Drowsiness – 272, Fever – 139, Irritability – 33, Loss of appetite – 36, Vomiting - 45	10.1016/S1473-3099(23)00519-4
nOPV2 + other vaccines	Primary	2 (2 week)	PerOs	1	293	N/D	N/D	N/D	N/D	11,5	1726	150,1	96,2	N/D	N/D	N/D	N/D	Abnormal crying – 90, Diarrhoea – 48, Drowsiness – 39, Fever – 144, Irritability – 45, Loss of appetite – 48, Vomiting - 75	10.1016/S1473-3099(23)00519-4
nOPV2 + other vaccines	Primary	2 (3 week)	PerOs	1	290	N/D	N/D	N/D	N/D	10,2	2328	228,2	97,2	N/D	N/D	N/D	N/D	Abnormal crying – 82, Diarrhoea – 39, Drowsiness – 36, Fever – 138, Irritability – 42, Loss of appetite – 39, Vomiting – 64; Fatal AE (3 months after the 2 dose): septic shock	10.1016/S1473-3099(23)00519-4
	Boost	2	PerOs	1	96	N/D	N/D	N/D	N/D	29,3	33,5	1,143	26,3	N/D	N/D	N/D	N/D	N/D	10.1016/S0140-6736(23)02844-1
bOPV2	Boost	1	PerOs	1	326	N/D	N/D	N/D	N/D	29,3	24,8	0,846	13,8	N/D	N/D	N/D	N/D	N/D	10.1016/S0140-6736(23)02844-1
tOPV	Primary	3 (2-3-4)	PerOs	1	296	7,8	2817	361,2	100	7,2	468,5	65,1	100	5,2	423,4	81,4	98,3	Bronchopneumonia -1, Epilepsy -1, Upper respiratory tract infection - 1, Bronchitis - 1, Enteritis -1 In one child - bronchopneumonia with abdominal distension, cardiac failure and respiratory failure	10.1016/j.vaccine.2016.02.010
tOPV	Primary	3 (2-3-4/3-4-5)	PerOs	1	78	9,48	1459,89	153,9	96,15	7,45	271,39	36,4	97,44	5,04	477,11	94,7	100	Fever - 170, Irritability - 15, Somnolence - 3, Vomit - 3, Diarrhea - 46, Allergic reaction - 2	10.1080/21645515.2017.1288769
IPV-tOPV-tOPV	Primary	3 (2-3-4/3-4-5)	IM/PerOs	1	86	8,27	1100,89	133,1	97,67	6,66	279,19	41,9	95,35	4,87	480,91	98,7	95,35	Fever - 186, Irritability - 10, Somnolence - 3, Vomit - 7, Diarrhea - 42, Redness on injection site - 1	10.1080/21645515.2017.1288769
IPV-IPV-tOPV	Primary	3 (2-3-4/3-4-5)	IM/PerOs	1	86	9,99	864,44	86,5	94,19	6,37	662,66	104	94,19	4,85	777,98	160,4	100	Fever - 202, Irritability/fussiness - 20, Somnolence - 2, Vomit - 14, Diarrhea - 46, Allergic reaction - 5, Redness on injection site - 4	10.1080/21645515.2017.1288769
IPV-bOPV-bOPV	Primary	3 (2-3-4/3-4-5)	IM/PerOs	1	86	11,27	1822,01	161,7	98,84	5,65	11,01	1,95	55,81	4,56	698,14	153,1	98,84	Fever - 173, Irritability - 13, Somnolence - 2, Vomit - 8, Diarrhea - 45, Allergic reaction - 1, Redness on injection site - 2	10.1080/21645515.2017.1288769
IPV-IPV-bOPV	Primary	3 (2-3-4/3-4-5)	IM/PerOs	1	86	10,87	1167,98	107,4	94,19	6,1	53,49	8,8	82,56	5,79	1068,89	184,6	97,67	Fever - 180, Irritability/fussiness - 20, Somnolence - 2, Vomit - 3, Diarrhea - 47, Allergic reaction - 5, Redness on injection site - 2	10.1080/21645515.2017.1288769
sIPV, bOPV, bOPV	Primary	3 (2-3-4)	IM/PerOs	1	189	7,35	5029,84	684,3	100	8,33	38,1	4,573	91,53	4,77	1406,72	294,9	100	Fever - 32, Rash - 9, Cough - 15, Diarrhea - 5, Running nose - 5, Allergy - 1, Redness - 3, Induration - 1, Swelling - 1	10.1016/j.vaccine.2020.07.042
sIPV, sIPV, bOPV	Primary	3 (2-3-4)	IM/PerOs	1	185	7,94	9310,89	1173	100	7,48	270,38	36,14	98,38	4,58	4065,43	887,6	100	Fever - 40, Rash - 9, Cough - 13, Diarrhea - 4, Running nose - 4, Crying - 1, Induration - 1	10.1016/j.vaccine.2020.07.042
DTwP-HB-Hib+bOPV+IPV	Primary	3 (2-4-6)	IM/PerOs	1	219	6,7	1516	226,3	100	8,11	53,2	6,6	98,2	5,97	1407	235,7	100	Tenderness - 202, Erythema - 100, Swelling - 89, Fever - 130, Vomiting - 67, Crying abnormal - 207, Drowsiness - 189, Appetite lost - 114, Irritability - 193	10.1097/INF.0000000000003975
DTwP-Hib + IPV + HBV	Primary	3 (6-10-14 W)	IM	1	111	11,7	585	50	98,2	16,7	795	47,6	98,2	6,7	774	115,5	100	Tenderness - 59, Erythema - 34, Swelling - 11, Fever - 12, Vomiting - 25, Crying abnormal - 36, Drowsiness - 41, Appetite lost - 23, Irritability - 51	10.1016/j.ijid.2011.10.002
DTwP-Hib + IPV +HBV	Primary	3 (6-10-14 W)	ID	/5	113	10,4	221	21,25	99,1	16,5	234	14,181	94,5	7,8	194	24,981	95,4	Tenderness - 71, Erythema - 82, Swelling - 25, Fever - 7, Vomiting - 18, Crying abnormal - 40, Drowsiness - 44, Appetite lost - 19, Irritability - 58	10.1016/j.ijid.2011.10.002
DTwP-HBV-IPV/Hib	Primary	3 (2-4-6)	IM	1	217	6,47	2272	351,2	100	9,1	2355	258,8	100	5,3	3040	573,6	100	Tenderness - 210, Erythema - 128, Swelling - 98, Fever - 157, Vomiting - 75, Crying abnormal - 206, Drowsiness - 183, Appetite lost - 141, Irritability - 196	10.1097/INF.0000000000003975
sIPV+DTPa	Primary	3 (2-3-4+5)	IM	1	170	5,55	369	66,48	95,29	5,32	153,43	28,8	96,47	4,73	237,07	50,12	100	Fever - 53, Irritability - 10, Vomit - 6, Diarrhea - 17, Drowsiness - 13, Eating disorder - 7, Allergic - 2, Pain - 9, Induration - 15, Redness - 64, Swelling - 8, Rash - 1, Skin - reactions 2	10.3389/fimmu.2022.905634
DTPa-IPV/Hib	Primary	3 (3-4-5)	SC	1	204	3,35	755	225,4	100	5,55	1090	196,4	100	2,86	719	251,4	100	Tenderness – 73, Erythema - 185, Swelling - 125 Induration - 152? Fever - 33, Vomiting - 42, Crying abnormal - 81, Drowsiness – 61, Loss of appetite - 47, Irritability - 113	10.1016/j.jiac.2019.11.012
DTPa-IPV/Hib	Primary	3 (3-4-5)	IM	1	10	6,96	549	78,9	100	9,85	923	93,7	100	3,61	724	200,6	100	Tenderness – 3 Erythema – 7, Swelling – 3, Induration – 6, Fever - 1, Vomiting - 1, Crying abnormal - 6, Drowsiness - 3, Loss of appetite 1, Irritability - 6	10.1016/j.jiac.2019.11.012
DTPa-IPV/Hib	Primary	3 (2-3-4)	IM	1	320	9,4	1143,7	121,7	ND	6,3	416,2	66,1	ND	5,8	1478,8	254,9	ND	Pain at the injection site – 90, Fever – 217, Irritability – 7, Nasopharyngitis – 42; Fatal AE (1 day post-dose 3): infectious shock due to acute bronchopneumonia and congestive heart failure	10.1080/21645515.2016.1239670
DTPa-IPV/Hib	Primary	3 (3-4-5)	IM	1	321	7,1	1328,9	187,2	ND	5	458,6	91,72	ND	4,9	1411,6	288,1	ND	Pain at the injection site - 91, Fever – 217, Irritability – 3, Nasopharyngitis – 46	10.1080/21645515.2016.1239670
DTPa-IPV/Hib	Primary	2 (2-4-6)	INJ	1	197	4,26	1237	290,4	ND	7,77	2081	267,8	ND	4,34	1868	430,4	ND	Tenderness – 127, Irritability – 106, Drowsiness – 102, Abnormal crying – 100, Fever – 33	10.3346/jkms.2016.31.9.1383
DTPa-IPV + Hib	Primary	3 (3-4-5)	SC	1	200	3,58	822	229,6	100	5,06	1158	228,9	100	2,95	810	274,6	100	Tenderness – 71, Erythema - 187, Swelling - 131, Induration - 149, Fever - 30, Vomiting - 38, Crying abnormal - 79, Drowsiness - 71, Loss of appetite - 40, Irritability - 111	10.1016/j.jiac.2019.11.012
DTPa-IPV + Hib	Primary	2 (2-4-6)	INJ	1	181	4,8	1042	217,1	ND	8,9	1781	200,1	ND	4,48	1557	347,5	ND	Tenderness – 119, Irritability – 110, Drowsiness – 104, Abnormal crying – 112, Fever - 23	10.3346/jkms.2016.31.9.1383
DTPa-IPV + Hib	Primary	3 (2-4-6)	IM	1	187	6,66	1218	182,9	100	9,72	3180	327,2	100	6,42	1920	299,1	100	Redness – 45, Swelling – 35, Pain – 25, Fever – 31, Drowsiness – 51, Fussiness – 49, Unusual crying – 45, Loss of appetite – 66, Vomiting – 23, Diarrhea - 26	10.1016/j.ijid.2005.10.014
DTPa-IPV –Hib	Primary	3 (2-4-6)	IM	1	100	6,07	1082	178,3	100	12,6	2504	198,7	100	7,6	1607	211,4	100	Redness – 44, Swelling – 32, Pain – 20, Fever – 21, Drowsiness – 37, Fussiness – 43, Unusual crying – 56, Loss of appetite – 70, Vomiting – 11, Diarrhea – 20	10.1016/j.ijid.2005.10.014
DTPa/Hib + IPV	Primary	3 (2-3-4)	IM	1	321	9,2	533,6	58	ND	6,9	186,4	27	ND	5,7	820,7	143,98	ND	Pain at the injection site – 102, Fever – 183, Irritability – 3, Nasopharyngitis - 50	10.1080/21645515.2016.1239670
DTPa-IPV/Hib*2+DTPa-HBV-IPV/Hib	Primary	3 (3-4-5)	IM	1	61	9,7	711,6	73,4	100	10,9	491,8	45,1	100	5,5	1606,8	292,1	100	Pain - 7, Redness - 9, Swelling - 12, Drowsiness - 5, Irritability - 14, Loss of appetite - 8, Temperature – 9	pubmed.ncbi.nlm.nih.gov/17987229/
DTPa-IPV/Hib*2+DTPa-IPV/Hib+HBV	Primary	3 (3-4-5)	IM	1	62	10	696,3	69,63	100	9,8	423,5	43,21	98,4	6,3	1138,8	180,7	98,4	Pain - 10, Redness - 12, Swelling - 5, Drowsiness - 15, Irritability - 19, Loss of appetite - 14, Temperature – 9	pubmed.ncbi.nlm.nih.gov/17987229/
DTPa-HBV-IPV/Hib	Primary	3 (6-10-14W)	IM	1	105	53,5	884,3	16,5	ND	32,5	840,2	25,9	ND	8	923,7	115,5	ND	Pain - 28, Fever - 17, Upper respiratory tract infection – 18	10.1080/21645515.2016.1225639
DTPa-HBV-IPV/Hib	Primary	3 (2-4-6)	IM	1	106	31,9	1799,2	56,4	ND	36,2	2138,7	59,1	ND	11,9	2245,5	188,7	ND	Pain - 15, Fever - 17, Upper respiratory tract infection – 11	10.1080/21645515.2016.1225639
DTPa-HBV-IPV/Hib+PCV13	Primary	3 (2-3-4)	IM	1	238	13,2	143,8	10,9	ND	18,8	81	4,3	ND	12,6	221,7	17,6	ND	Irritability - 192, Fever - 141	10.1080/21645515.2017.1294294
DTPa-HBV-IPV/Hib (form. A)+PCV13	Primary	3 (2-3-4)	IM	1	238	13,5	110	8,15	ND	16	72	4,5	ND	13	179,4	13,8	ND	Irritability – 197, Fever – 180; Fatal AE (17 days post-dose 1): asphyxia and interstitial lung disease	10.1080/21645515.2017.1294294
DTPa-HBV-IPV/Hib (form. B)+PCV13	Primary	3 (2-3-4)	IM	1	239	13,3	94,6	7,1	ND	18,4	68,5	3,7	ND	12,5	159,6	12,8	ND	Irritability – 207, Fever – 174	10.1080/21645515.2017.1294294
IPV + RotaV5 (concomitant)	Primary	3 (2-3-4)	IM	1	180	15,22	5600,8	367,9	98,9	9,76	1059,8	108,6	98,3	5,88	3405,56	579,2	100	Vomiting - 20, Diarrhea - 33, Dyspepsia - 10, Erythema - 45, Pain - 12, Pyrexia - 30, Bronchitis - 11, Nasopharyngitis - 14, Upper respiratory tract infection - 31, Cough - 19, Rhinorrhea - 17, Eczema - 14	10.1080/21645515.2024.2324538
IPV + RotaV5 (sequential)	Primary	3 (2-3-4)+RV5 in 1 Mo	IM	1	187	15,66	5344,24	341,3	100	8,86	1122,4	126,7	99,5	6,49	3261,69	502,6	99,5	Vomiting - 39, Diarrhea - 50, Dyspepsia - 13, Injection site erythema - 41, Injection site pain - 7, Pyrexia - 37, Bronchitis - 13, Nasopharyngitis - 14, Upper respiratory tract infection - 37, Cough - 14, Rhinorrhea - 18, Eczema - 12	10.1080/21645515.2024.2324538
